# Editorial: Immunological Challenges Following Pediatric Hematopoietic Transplantation

**DOI:** 10.3389/fimmu.2021.732261

**Published:** 2021-07-20

**Authors:** Robert J. Hayashi

**Affiliations:** Division of Pediatric Hematology/Oncology, Washington University School of Medicine, St. Louis Children’s Hospital, St. Louis, MO, United States

**Keywords:** hematopoietic stem cell transplantation, immunology, pediatrics, non-malignant disease, regulation

Advancements in hematopoietic stem cell transplantation (HSCT) has made this treatment modality a viable option for hematopoietic based diseases. With the expansion of donor options and the reduced toxicities of preparative regimens, the risk/benefits of HSCT are becoming more and more favorable, making it preferable than to face the complications of a patient’s primary disease. Opportunities to improve upon the transplant procedure centrally weighs on improving our understanding of the immune system as most of the primary obstacles for success lie within the immunologic challenges either from the host or the donor. Whether it is overcoming the threats of rejection, the complications of excessive immunosuppression leading to infections, or post-transplant lymphoproliferative disease, (PTLD), or the emergence of immune dysregulation leading to autoimmunity or graft versus host disease, achieving the full potential of this treatment modality rests on our ability to safely eradicate the pre-existing immune system and to establish a competent, regulated one from the donor cells. The pathway to success rests on our ability to sustain 1) Hematopoietic engraftment, 2) Immunologic competence, and 3) Donor cell tolerance (**Figure 1**). Failure to maintain all three will invariably lead to life threatening complications. The collection of manuscripts for this Research Topic spans the full scope of immunological challenges that lay before us, providing insights on what future investigations are needed to overcome them.

**Figure 1:**
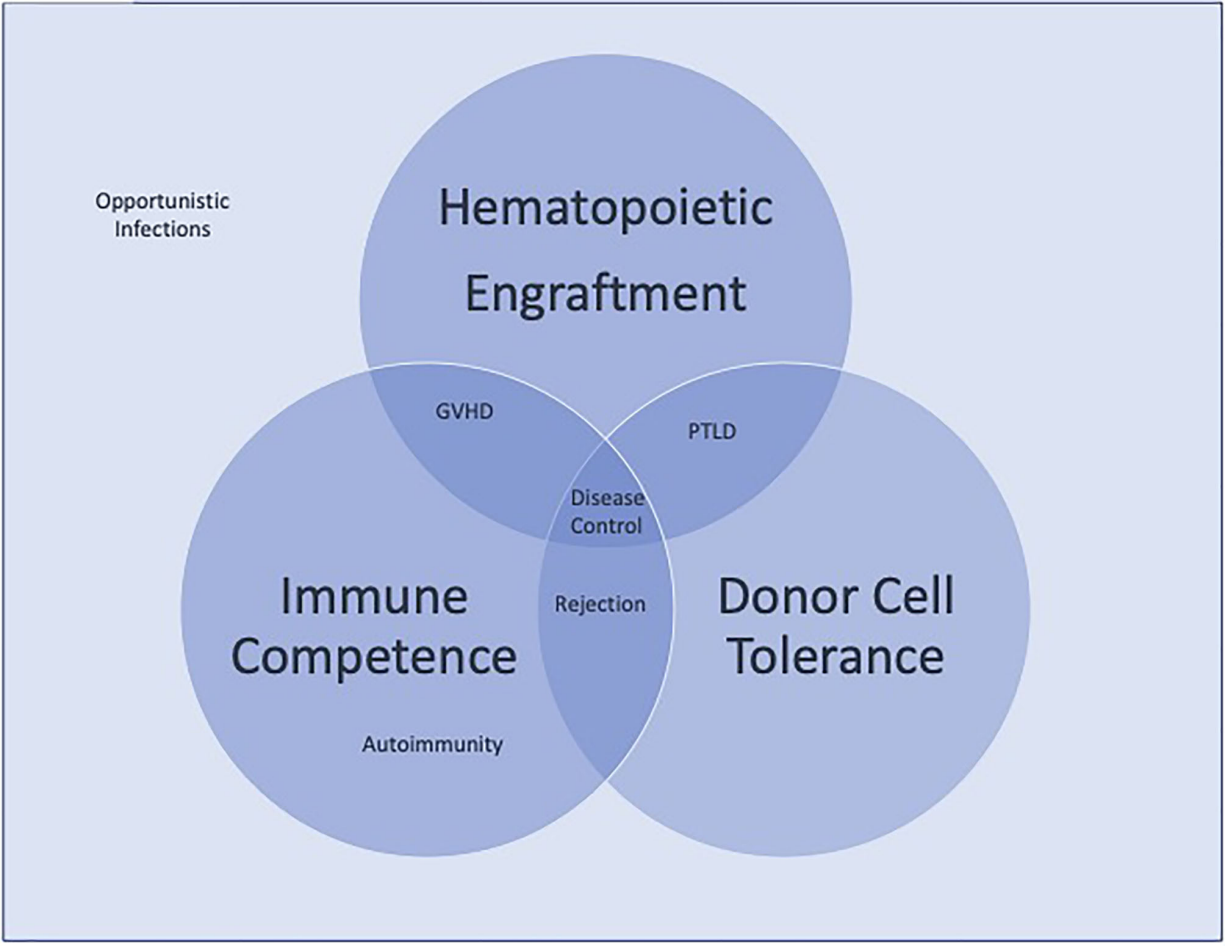
Competing immunologic forces in hematopoietic stem cell transplantation. Disease control requires the balance of hematologic engraftment, immunologic competence, and donor cell tolerance. Failure to achieve all three leads to the clinical complications of transplantation represented.

Establishing engraftment and minimizing long term toxicity necessitates a thoughtful approach in the selection of the preparative regimen The repertoire of agents for consideration are reviewed in this Research Topic (Hayashi), and although the correct selection invariably differs with the disease of interest, the optimal regimen for most conditions has yet to be defined. Further complicating the issue is the degree of chimerism required to establish a curative outcome for a particular disease. As discussed by Zimmerman and Shenoy, the lack of the necessity for complete donor chimerism to provide efficacy for some diseases gives the clinician flexibility to refine preparative regimens to establish minimum level of donor engraftment to achieve disease control. Challenges remain with our lack of understanding of not only the degree of engraftment needed for each disease but also the variables that ensure stable engraftment in a partial chimera state.

Establishing a new immune system with donor engraftment requires a keen awareness of the essential elements of immune reconstitution along with the vulnerabilities the host experiences to different pathogens at different time points as the new immune system is generated. The elements of establishing a robust donor immune system rather than one which leads to infectious risks and rejection was reviewed by Bhatt and Bednarski. A prolonged incompetent immune system leads to susceptibility to viral pathogens and can also lead to additional complications such as PTLD. Our current struggles in promptly establishing immune competence in the post-transplant period incentivizes us to pursue alternative strategies to protect the patient, harnessing our knowledge of effector mechanisms to aid host defenses until sufficient immune reconstitution is achieved. As summarized in Basso et al.’s review, means of generating anti-pathogen effector cells are being developed *via* a variety of strategies and the optimization of such therapies will be a substantial advancement in the battle against infections where effective antibiotics may be lacking. Such efforts can also be utilized to combat Epstein Barr virus driven disease processes such as PTLD (Compagno et al.).

Once donor immunity establishes itself, the threats of rejection and immunodeficiency are supplanted by the threat of dysregulated immunity. As reviewed by Buxbaum and Pavletic, most autoimmune processes are B cell mediated and can be a consequence from residual donor B cells, or donor cells responding to host antigens with dysregulated T cells. In contrast, chronic graft versus host disease is much more complex complication, recruiting all elements of the immune system.

Chronic graft *versus* host disease remains one of the most debilitating and life threatening immune mediated complication of the transplant process. Fully elucidating the mechanisms and identifying targetable elements can provide opportunities to improve outcomes. Rozmus’ suggestion that the study of monogenic diseases may give us novel insights in identifying new targets against graft *versus* host disease is a provocative one, and pursuit of investigations along this strategy will hopefully provide new therapeutic opportunities in a disease in need of new treatments. Increasing our understanding of how the elements of the immune system is organized in chronic graft versus host disease is also of critical importance to formulate thoughtful treatment strategies. Cuvelier et al.’s manuscript that the cells responsible for chronic graft versus host disease differ between adults and children highlights a paradigm that requires further study. This observation suggests that the challenges that we must overcome to understand this disease are even more formidable that what we have traditionally thought; and future efforts must take age related issues into account if we are going to improve transplant outcomes for the pediatric population.

Still, optimism exists, as novel therapies continue to emerge with time. Ringden et al.’s report of their experience using mesenchymal stem cells to treat steroid refractory graft *versus* host disease illustrates the wide scope of therapeutic avenues that are being explored to find impactful therapies for this challenging condition.

Thus, it is clear that there remain many immunologic challenges that need to be overcome to improve the outcomes of HSCT for pediatric non-malignant disease. This collection of reports provides clarity, not only on where we are in this journey, but also highlights potential pathways for success. The pace by which we gain greater command of transplant immunology will dictate the pace in which HSCT becomes the primary therapeutic choice in the treatment of hematopoietic diseases.

## Author Contributions

The author confirms being the sole contributor of this work and has approved it for publication.

## Conflict of Interest

The author declares that the research was conducted in the absence of any commercial or financial relationships that could be construed as a potential conflict of interest.

